# Perforation iléale iatrogène: complication mécanique exceptionnelle de la pose du cathéter d’hémodialyse au niveau du site fémoral

**Published:** 2010-06-27

**Authors:** Abderrahim El Bouazzaoui, Ali Darkaoui, Nawfal Houari, Karima Attaraf, Youssef Bouabdellah, Smael Labib, Mustapha Harandou

**Affiliations:** 1 Service réanimation mère et enfant; 2 Service de chirurgie viscérale infantile, Hôpital mère et enfant, CHU Hassan II- Fès, Maroc

**Keywords:** Perforation iatrogène, cathéter, hémodialyse, Maroc

## Abstract

Le cathétérisme veineux fémoral est d’indication fréquente et de réalisation le plus souvent aisée et rapide. Thrombose et infection sont les complications les plus couramment rapportées. La perforation iatrogène d’une anse digestive par le trocart de ponction lors de la mise en place d’un cathéter d’hémodialyse au niveau du site fémoral  est une complication mécanique inhabituelle. Nous en rapportons un cas jamais décrit, à notre connaissance, dans la littérature médicale.

## Introduction

Quand l’hémodialyse est indiquée en urgence, la veine fémorale est le site le plus simple et le plus facile pour l’insertion du cathéter d’hémodialyse [1,2]. Selon une étude menée par El Minshawy et coll, les complications communes sont marquées par les infections dans 40 % des cas, les thromboses dans 7.5 %, saignement d’une artère fémorale perforée dans 5 % et l’hématome de l’aine dans 0.5 % [2]. D’autres rares complications mécaniques ont été décrites telles que la fistule artérioveineuse fémorale, l’hématome rétro péritonéal, l’abcès du muscle psoas et l’hématome pré vésical [3-6]. La perforation iatrogène d’une anse digestive par le trocart de ponction lors de la mise en place d’un cathéter d’hémodialyse au niveau du site fémoral est une complication mécanique exceptionnelle. Nous en rapportons un cas jamais décrit, à notre connaissance, dans la littérature médicale.

## Patient et observation

**Figure 1:**
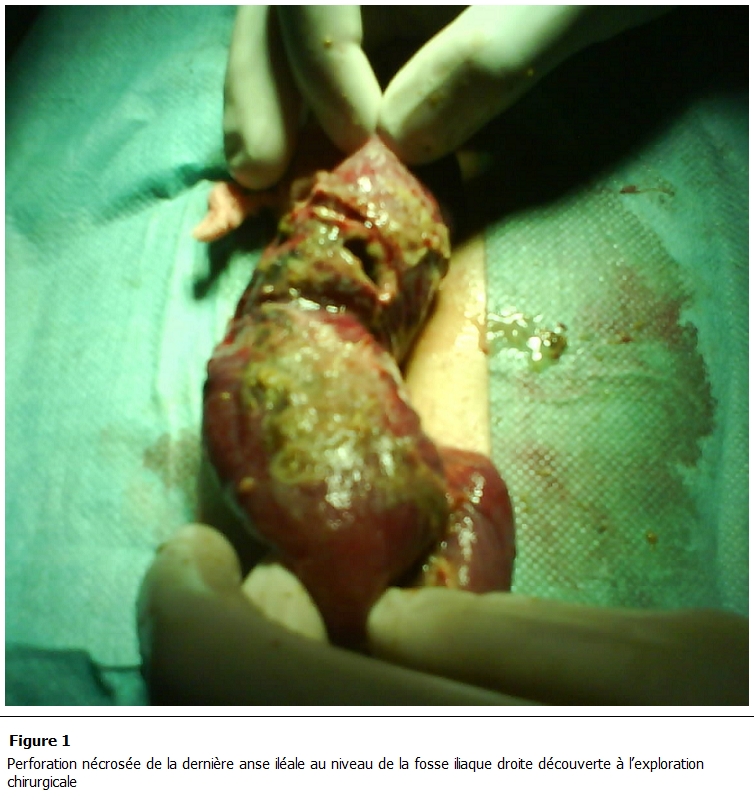
Perforation nécrosée de la dernière anse iléale au niveau de la fosse iliaque droite découverte à l’exploration chirurgicale

Fillette de douze ans, sans antécédents particuliers, présente depuis un an des coliques néphrétiques gauches, évoluant dans un contexte d’altération de l’état général. Ce tableau s’est aggravé, deux semaines avant son admission, par l’apparition d’un syndrome fébrile. L’examen à son admission révèle une fillette cachectique, pesant 35 kg (- 2 DS), taille 1,30 m, TA = 160/100 mmHg, conjonctives décolorées avec un teint grisâtre. L’abdomen est souple, sans contact lombaire. L’échographie rénale a montré un rein gauche en fer à cheval, avec une atrophie corticale. Le bilan biologique est revenu en faveur d’une insuffisance rénale terminale (urée= 2,3 g/L, créatinémie =65 mg/l avec une clairance de la créatinine à 8.13 ml/min. Une hémodialyse a été donc indiquée par les néphrologues et un cathéter d’hémodialyse bilumière pédiatrique est inséré au niveau de la veine fémorale droite. Les repères anatomiques sont respectés lors de ce geste, les tentatives de pose sont au nombre de quatre et la technique adoptée est celle de Seldinger. Deux jours plus tard, la patiente présente un syndrome occlusif. Une échographie abdominale met en évidence un épanchement intra péritonéale de moyenne abondance. Le scanner abdominal objective un aspect du volvulus du colon sigmoïde. A l’exploration chirurgicale on découvre une perforation de la dernière anse iléale au niveau de la fosse iliaque droite. Cette perforation nécrosée (Figure 1), colmatée par l’épiploon et le reste du grêle, est accolée à la paroi abdominale inférieure. On réalise alors une toilette péritonéale et une résection-stomie. L’évolution est malheureusement défavorable vers un tableau de choc septique sur une péritonite postopératoire entraînant le décès de la patiente cinq jours plus tard, malgré une antibiothérapie adaptée et une réanimation médicale spécialisée.

## Discussion

En urgence, lorsqu’un accès veineux central est nécessaire, la voie fémorale est recommandée [7], car elle permet un excellent débit de perfusion lorsque le cathéter est de diamètre suffisant et est facile d’accès [7-9]. Les complications initiales sont représentées par des ponctions artérielles (9–15 %) et des hématomes (4 %), le plus souvent bénins [10].

Jusqu’à une date récente, le choix de ce site avait été une technique insuffisamment utilisée pour l’accès à la circulation centrale, vraisemblablement en raison d’une incidence élevée des complications infectieuses et thrombotiques rapportées dans la littérature médicale [11]. Cependant, des études plus récentes ont suggéré que ce site ne présente pas un taux élevé de complication infectieuse ou thrombotiques que les autres sites [12]. La veine fémorale est maintenant identifiée comme emplacement favorable pour le cathétérisme veineux central en raison d’un indice de réussite élevé et une incidence plus limitée des complications sérieuses mettant en jeu le pronostic vital [12].

La veine fémorale est habituellement ponctionnée au niveau du triangle de Scarpa, au-dessous de l’arcade crurale, tendue entre l’épine iliaque antérosupérieure et l’épine du pubis.

L’enfant est installé en décubitus dorsal en léger proclive avec un billot sous le bassin pour permettre une bonne extension de la hanche, le membre inférieur placé en rotation externe. Après une détersion et une asepsie rigoureuse, la ponction doit être réalisée 1 à 2 cm sous l’arcade crurale. L’aiguille doit être dirigée vers le haut dans l’axe vasculaire à 45° en profondeur [13].

La pose de voies veineuses centrales se fait habituellement par ponction percutanée à l’aveugle aidée par la connaissance de l’anatomie habituelle des vaisseaux, l’existence de repères cutanés ou osseux et, le cas échéant, la palpation d’une artère de voisinage (carotide pour la jugulaire interne, artère fémorale pour la veine fémorale) [14].

Selon une étude menée par David et all, plus de 15 % des malades ayant bénéficié d’un cathétérisme veineux central présentent au moins une complication. Ces complications sont d’ordre infectieux dans 5 à 26%, thrombotiques dans 2 à 26%, ou mécaniques dans 5 à 19% des cas [15].

Au niveau du site fémoral, les accidents mécaniques sont dominés essentiellement par l’hématome et la ponction artérielle [16]. Des auteurs ont rapporté des cas de complications mécaniques rares, voire exceptionnelles du cathétérisme veineux central fémoral. Nous en citons le cas de l’hémopéritoine iatrogène lors de la ponction [9] ou encore le cas de l’hématome retro péritonéal massif décrit par Takashi et all [12]. J Berrada a rapporté le cas de la migration du désilet métallique dans la lumière de la veine fémorale [17]. Finalement nous citons le cas de la perforation vasculaire tardive dans le territoire cave inferieure rapporté par C Gil at all [18].

L’expérience de l’opérateur joue, certes, un rôle dans la survenue de ces complications mécaniques. Ainsi, Sznajder démontre dans un écrit que lorsqu’un opérateur effectue plus de 50 poses de cathéter veineux central, les complications mécaniques diminuent d’environ 50 % [19]. Autrement, Eisen et collaborateurs démontrent que lorsqu’un opérateur dépasse deux tentatives de pose, le risque de survenue d’une complication mécanique passe de 17% à 54 % [16].

La connaissance et le respect des repères anatomiques sont donc primordiaux pour éviter les complications surtout d’ordre mécanique. Mais, malheureusement, la technique classique du repérage anatomique n’est pas exempte de complications, lesquelles contribuent à une augmentation des durées et des coûts d’hospitalisation et majorent le risque de survenue de complications secondaires [20]. Pour limiter l’incidence de ces complications immédiates, des équipes anglo-saxonnes ont prôné l’usage de l’échographie bidimensionnelle [14]. Le bénéfice de ce guidage ultrasonographique pour l’abord veineux central est aujourd’hui bien établi chez l’adulte comme chez l’enfant [21,22]. Ainsi, s’appuyant sur une méta-analyse récente [21], le National Institute for Clinical Excellence (NICE) britannique a diffusé des recommandations suivantes [23]: 1) Le guidage de la ponction par l’échographie bidimensionnelle est recommandé lors de la mise en place des cathéters veineux centraux par voie jugulaire interne chez l’adulte et chez l’enfant; 2) dans les situations d’urgence, la possibilité de recourir aux ultrasons devrait être considérée; 3) une formation appropriée des professionnels susceptibles de placer des cathéters veineux centraux sous écho-guidage est recommandée; 4) l’utilisation du doppler sonore comme mode de guidage n’est pas recommandée.

L’avantage des techniques de repérage échographique de la veine concerne essentiellement la veine jugulaire interne, les données concernant la veine sous-clavière ou la veine fémorale étant plus limitées [24].

La généralisation de cette technique devrait être favorisée par la mise à la disposition des opérateurs d’appareils portatifs de coût réduit et par la facilité d’apprentissage de la technique [24]. Malgré ces recommandations, le coût et l’apprentissage probablement nécessaires de ces techniques restent encore un obstacle à leur généralisation.

Dans notre cas, nous estimons que la taille du trocart n’était pas adaptée à la corpulence de l’enfant, et que la ponction de la veine fémorale a été réalisée au dessus de l’arcade crurale. Ce cathétérisme de la veine fémorale a été réalisé par un jeune résident en formation au cours d’une garde, et en l’absence du senior. Cette perforation iléale passée inaperçue ne s’est manifestée que tardivement par un syndrome occlusif sur une perforation iléale bouchée.

Cette complication mécanique rare jamais décrite – à notre connaissance – souligne l’intérêt du respect de certaines règles telles que l’expérience du médecin, le respect des repères anatomiques et le guidage ultrasonographique.

## Conclusion

Nous rappelons que si l’accès veineux fémoral est généralement facile, il n’est pas dénué de complications mécaniques potentiellement graves. Le respect des repères anatomiques, l’expérience du praticien et le guidage ultrasonographique sont des règles à respecter. La réduction de ces complication implique une parfaite maîtrise de la technique et l’application d’une démarche qualité incluant procédures écrites et formation des acteurs.

## Consentement

On a eu le consentement de la patiente. De ce fait la publication de cette observation ne pose aucun problème pour notre patiente ou bien pour ses proches.

## Contribution des auteurs

Tous les auteurs ont contribué à la réalisation et à la description de cette observation médicale. Les auteurs relevant du service d’anesthésie ont vécu le cas au cours d’une garde. Ils ont fait une recherche bibliographique et ont rédigé cette modeste observation. L’équipe chirurgicale s’est occupée du coté chirurgical.
